# Human NK cell lytic granules and regulation of their exocytosis

**DOI:** 10.3389/fimmu.2012.00335

**Published:** 2012-11-09

**Authors:** Konrad Krzewski, John E. Coligan

**Affiliations:** Receptor Cell Biology Section, Laboratory of Immunogenetics, National Institute of Allergy and Infectious Diseases, National Institutes of HealthRockville, MD, USA

**Keywords:** NK cells, cytotoxic lymphocytes, lytic granules, lysosomes, cytotoxicity, exocytosis

## Abstract

Natural killer (NK) cells form a subset of lymphocytes that play a key role in immuno-surveillance and host defense against cancer and viral infections. They recognize stressed cells through a variety of germline-encoded activating cell surface receptors and utilize their cytotoxic ability to eliminate abnormal cells. Killing of target cells is a complex, multi-stage process that concludes in the directed secretion of lytic granules, containing perforin and granzymes, at the immunological synapse. Upon delivery to a target cell, perforin mediates generation of pores in membranes of target cells, allowing granzymes to access target cell cytoplasm and induce apoptosis. Therefore, lytic granules of NK cells are indispensable for normal NK cell cytolytic function. Indeed, defects in lytic granule secretion lead or are related to serious and often fatal diseases, such as familial hemophagocytic lymphohistiocytosis (FHL) type 2–5 or Griscelli syndrome type 2. A number of reports highlight the role of several proteins involved in lytic granule release and NK cell-mediated killing of tumor cells. This review focuses on lytic granules of human NK cells and the advancements in understanding the mechanisms controlling their exocytosis.

## NK cells and their activation for lytic granule polarization

Natural killer (NK) cells comprise a subset of lymphocytes involved in protection against microbial pathogens and tumors (Orange, 2006; Vivier et al., 2008). Initially considered to be only an integral part of the innate immune response, they have been later shown to be involved in modulation of the adaptive immune response, by virtue of chemokine and cytokine production (e.g., interferon γ, TNFα, MIP-1α/β, and RANTES), and regulation of activity of other cells of the immune system, such as dendritic cells, macrophages or T cells (Fernandez et al., 1999; Gerosa et al., 2002; Robertson, 2002; Cooper et al., 2004; Orange and Ballas, 2006; Fauriat et al., 2010).

NK cells express a variety of germline-encoded inhibitory or activating cell surface receptors [reviewed in detail in (Chiesa et al., 2005; Lanier, 2005; Watzl and Urlaub, 2012)]. NK cell activating receptors recognize ligands expressed by abnormal cells and, in the majority of cases, convey the signal through interaction with signaling adaptors. For example, the activating receptors CD16, NKG2C/CD94, or natural cytotoxicity receptors NKp30, NKp44, and NKp46 interact with immunoreceptor tyrosine-based activating motif (ITAM)-bearing polypeptides (i.e., CD3ζ, DAP12, FcRγ), while other receptors, such as NKG2D or 2B4, bind to non-ITAM-bearing proteins (e.g., DAP10, SAP) (Chiesa et al., 2005; Lanier, 2005; Bryceson et al., 2006a). Because some of the ligands for activating receptors can be expressed by normal cells, NK cells also express a panel of inhibitory receptors that, in majority, recognize MHC class I molecules expressed by most cells. Inhibitory receptors are characterized by the presence of cytoplasmic immunoreceptor tyrosine-based inhibition motifs (ITIMs) and, generally, belong to one of the two groups of molecules: immunoglobulin superfamily (e.g., KIR, LIR, siglecs) or C-type lectin domain family (e.g., NKG2A/CD94) (Chiesa et al., 2005; Lanier, 2005; Bryceson et al., 2006a; Long, 2008). The density of the inhibitory receptors (or their ligands) on the cell surface influences NK cell cytotoxicity; a certain level of inhibitory receptor expression is required for restriction of cytotoxicity (Almeida and Davis, 2006; Endt et al., 2007; Almeida et al., 2011). In normal physiological conditions, the number of inhibitory receptors as well as the density of their ligands on the cell surface is sufficient to maximally engage inhibitory receptors (Almeida and Davis, 2006; Endt et al., 2007). When both activating and inhibitory receptors are co-engaged at the same time, signals from the inhibitory receptors override activation signals, thereby preventing destruction of normal, healthy cells (Watzl et al., 2000; Masilamani et al., 2006; Endt et al., 2007; Bryceson et al., 2009). On stressed cells, however, ligands for the inhibitory receptors tend to be down-regulated and/or activating receptor ligands are up-regulated, thereby favoring signaling through activation receptors. Thus, the balance between signaling of different receptor groups regulates the activity of NK cells.

The activation and/or inhibition signals are generated at the specialized contact site formed between the NK and target cell, known as the immunological synapse. The immunological synapse is defined as a contact between two cells, at least one of them being the immune system cell (e.g., NK cell), that results in the segregation of proteins at the cell-cell interface into micrometer-scale three-dimensional domains (Davis, 2002; Krzewski and Strominger, 2008; Orange, 2008). A unique feature of human NK cells is the fact, that depending on the target cell and NK cell receptor ligands being recognized, NK cells are able to form many types of immunological synapses, such as NK cell activating, inhibitory, or regulatory synapses. Those synapses play specialized functions and vary in the spatial organization between themselves, and may differ from immunological synapses formed by other cells of the immune system, and are reviewed elsewhere (Krzewski and Strominger, 2008).

**Figure 1:**
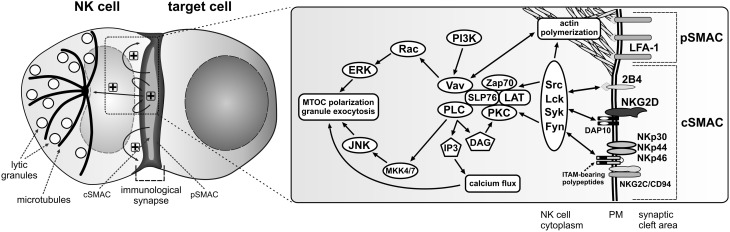
**Activation signals for lytic granule polarization in NK cells.** The encounter between an NK cell and a susceptible target cell results in conjugation and formation of the activating immunological synapse. Adhesion molecules, such as LFA-1, segregate into the outer region of the synapse, referred to as the peripheral supramolecular activation cluster (pSMAC), while NK cell activating receptors localize into the central area of the synapse (cSMAC). Engagement of NK cell activating receptors by their ligands on the target cell (not shown) induces phosphorylation of membrane proximal signaling molecules and formation of a signalosome comprised of many signaling and adapter molecules at the cSMAC. Positive feedback loops are generated, causing signal amplification and sustained signaling (“+” symbols) that stimulates more robust actin polymerization at the synapse periphery and polarization of the MTOC and lytic granules to the immunological synapse, where the granules will be subsequently exocytosed. The diagrams represent only selected molecules. The drawings are not to scale.

The interaction between an NK cell and a target cell having diminished or lacking appropriate ligands for the NK cell inhibitory receptors usually results in generation of an activating NK cell immunological synapse (Figure 1). It is a complex and tightly controlled process involving multiple steps, leading to release of cytolytic granules and subsequent death of the target cell (Krzewski and Strominger, 2008; Orange, 2008). Following the contact with the susceptible target cell, adhesion molecules, such as LFA-1 (CD11a/CD18) and Mac-1 (CD11b/CD18), segregate into an outer region of the synapse, known as the peripheral supramolecular activation cluster (pSMAC) (Vyas et al., 2001; Orange et al., 2003; Liu et al., 2009), where they mediate formation of a tight conjugate between the cells, and play important roles in generation of initial activation cues. For instance, LFA-1 signaling is required for phosphorylation of several signaling molecules, including Src, LAT, SLP76, ZAP70, Vav-1, PKCs, ERK1/2, or JNK (Riteau et al., 2003; Perez et al., 2004; Chen et al., 2006). Signals coming from LFA-1 induce actin polymerization and accumulation of filamentous actin at the pSMAC (Orange et al., 2003; Chen et al., 2007; Krzewski et al., 2008), and are important for lytic granule polarization to the immunological synapse (Barber et al., 2004; Perez et al., 2004; Bryceson et al., 2005). Other molecules that accumulate at the pSMAC and contribute to actin polymerization include talin, ezrin-radixin-moesin proteins, and WASp (Vyas et al., 2001; Orange et al., 2002; Ramoni et al., 2002; McCann et al., 2003; Roda-Navarro et al., 2004).

While the adhesion and co-stimulatory molecules localize to the pSMAC, the activating receptors accumulate in a middle area of the immunological synapse, known as the central supramolecular activation cluster (cSMAC) (Vyas et al., 2001, 2002) (Figure 1), where their synergistic signaling is fundamental to facilitate cytotoxic responses (Bryceson et al., 2006a,b, 2009; Kim et al., 2010). The engagement of activating receptors leads to recruitment of molecules integral in signal transduction: Src, Lck, Syk, Fyn, ZAP70, and PKC kinases, scaffolding proteins like SLP76, LAT, BLNK, as well as other signaling molecules such as Vav-1, Grb2, PLCγ, PI3K, Pyk2, Rap1, or CrkL (Sancho et al., 2000; Vyas et al., 2001, 2002; Riteau et al., 2003; Upshaw et al., 2006; Segovis et al., 2009). This, in turn, causes activation of two major pathways involved in cytolytic responses: PI3K–ERK2 (Jiang et al., 2000; Chen et al., 2006, 2007) and PLCγ–JNK (Li et al., 2008) (Figure 1).

The synergistic action of human NK cell activating receptors and initiation of different signaling cascades serves to stimulate actin polymerization at the cell-cell contact site, and subsequent translocation of the microtubule organizing center (MTOC) with associated lytic granules toward the immunological synapse. The disorganization of the actin cytoskeleton, either as a result of an immune disorder like the Wiskott-Aldrich syndrome or use of pharmacological agents, results in severe impairment of NK cell activity (Orange et al., 2002, 2003; Wulfing et al., 2003; Barber et al., 2004). Disruption of expression of proteins involved in actin cytoskeleton rearrangements, for instance Arp2/3, formins, HS1, WASp, or WIP, negatively affects NK cell cytotoxicity by blocking NK cell adhesion, granule transport and/or exocytosis (Orange et al., 2002, 2003; Krzewski et al., 2006, 2008; Butler et al., 2008; Butler and Cooper, 2009). Likewise, compromising the integrity of microtubules blocks granule polarization to the immunological synapse (Orange et al., 2003; Mentlik et al., 2010). Furthermore, silencing of CIP4, a protein that links microtubules and the actin network through its ability to bind microtubules and WASp, severely impairs the MTOC polarization and NK cell cytotoxicity (Banerjee et al., 2007). Interfering with function of IQGAP1, a protein involved in cytoskeletal rearrangements and bridging actin cytoskeleton with microtubule plus-end-tracking proteins (e.g., CLIP-170, APC), results in the inability of NK cells to translocate the MTOC toward the immunological synapse (Kanwar and Wilkins, 2011). Similarly, knock-down of formin hDia1, or its microtubule-associated effector proteins, APC and EB-1, blocks the recruitment of microtubules and the MTOC to the human NK cell activating synapse (Butler and Cooper, 2009). Thus, the integrity of the cytoskeleton is imperative for NK cell function and exocytosis of lytic granules.

## Lytic granules and their content

Lytic granules are often referred to as “secretory lysosomes,” due to the fact that they have the characteristics of lysosomes (Burkhardt et al., 1990; Peters et al., 1991; Blott and Griffiths, 2002). Their content is separated from the cytoplasm by a bi-layer membrane, and is composed of a variety of enzymes typical for lysosomes, as well as proteins unique to lytic granules. The latter are represented by perforin, granzymes, Fas ligand (FasL; CD178), TNF-related apoptosis-inducing ligand (TRAIL; CD253), granulysin, and small anti-microbial peptides (see below). By electron microscopy, the lytic granules of NK cells appear to be relatively heterogeneous, with three distinguishable classes: type I, type II and intermediate granules (Neighbour et al., 1982; Burkhardt et al., 1990). Type I granules (50–700 nm) are mostly filled by a dense core surrounded by thin layer of vesicles, while type II granules (200–1000 nm) are characterized by multiple vesicles and membrane whorls. The intermediate granules have both dense cores, although smaller than type I granules, and multiple vesicles that are not as abundant as in type II granules. Likely, type I granules are fully developed lytic granules, with type II and intermediate forms representing different stages of granule development (Neighbour et al., 1982). The multivesicular bodies of lytic granules contain cathepsins and other lysosomal hydrolases (e.g., α-glucosidase, acid phosphatase), and generally are devoid of the cytotoxic enzymes; the dense cores of lytic granules contain lattices of the chondroitin-sulfate proteoglycan serglycin (that gives the cores their characteristic electron density), as well as perforin and granzymes (Burkhardt et al., 1990).

### Granzymes

Granzymes belong to a group of serine proteases. To date, five granzyme genes organized in three clusters have been identified in humans (Grossman et al., 2003) and found to be constitutively transcribed in NK cells (Salcedo et al., 1993). Granzyme A cluster, located on chromosome 5, encodes granzyme A and K. Granzyme B cluster, on chromosome 14, contains genes for granzyme B and H. Granzyme M cluster, found on chromosome 19, encodes granzyme M. Granzyme A and K cleave their substrates after arginine or lysine residues, and thus are tryptases; granzyme B (Asp-ase) cleaves after aspartic acid residue, granzyme H (chymase) cleaves substrates at hydrophobic residues (Phe, Tyr or Trp), and granzyme M (Met-ase) cleaves at methionine or leucine residues (Poe et al., 1991; Edwards et al., 1999; Kam et al., 2000; Grossman et al., 2003). Granzymes are synthesized as pro-enzymes and reach the lytic granules mainly through the mannose-6-phosphate receptor (MPR) pathway (Griffiths and Isaaz, 1993). In the granules, granzymes are processed to an active form by cathepsin C (and H, in the case of granzyme B) (Smyth et al., 1995; Meade et al., 2006; D'Angelo et al., 2010), and are packed tightly through complexing with serglycin (Metkar et al., 2002; Raja et al., 2002).

### Perforin

Perforin is a multi-domain, pore-forming protein (Voskoboinik et al., 2006, 2010). The perforin gene is constitutively transcribed in NK cells (Nakata et al., 1992; Salcedo et al., 1993); however, the CD56^bright^ NK cells have lower levels of perforin than the more mature CD56^dim^ NK cells. The protein is synthesized as a 555 amino-acid, 65 kDa precursor in the ER. The N-terminus of perforin contains a signal peptide and the membrane attack complex/perforin (MACPF) domain, and is known to have lytic activity (Rochel and Cowan, 1996). The C-terminal part incorporates an EGF-like domain and a C2 domain that is able to bind membranes in a calcium-dependent manner (Voskoboinik et al., 2005a). The extreme C-terminus (past the C2 domain) is required for the efficient transport of perforin from the ER to the Golgi apparatus, while the N-glycosylation sites are needed for trafficking of perforin from the Golgi to lytic granules (Brennan et al., 2011). How exactly perforin reaches lytic granules after leaving the TGN is not fully elucidated. A possible sorting mechanism could involve the MPR-dependent pathway, as recently postulated (Brennan et al., 2011). Although perforin has been reported to lack mannose-6-phosphate (M6P) modification (Burkhardt et al., 1989), it is possible for lysosomal proteins to use MPR-positive carrier vesicles even though they do not have the modification; for example, neuraminidase uses the interaction with M6P-modified cathepsin A to reach lysosomes (van der Spoel et al., 1998). In addition, it is not uncommon for lysosomal proteins to use different pathways to reach lysosomes: a portion of lysosomal enzymes that normally use the MPR pathway are still sorted to lysosomes in cells from I-cell disease patients that do not have a functional MPR pathway, and even with both glycosylation sites mutated, a fraction of perforin is still able to reach granules (Glickman and Kornfeld, 1993; Griffiths and Isaaz, 1993; Brennan et al., 2011). After delivery to the lytic granules, perforin is subject to proteolytic cleavage by cathepsin L that removes the last 20 aa at the C-terminal part of perforin, and is required for full perforin activity (Uellner et al., 1997; Konjar et al., 2010). In the lytic granules, perforin activity is inhibited by virtue of a combination of several factors, such as an acidic environment of granules, presence of calreticulin and calreticulin-mediated increased resistance of granule membranes to osmotic lysis, as well as interaction with serglycin (Fraser et al., 2000; Metkar et al., 2002).

### FasL and TRAIL

FasL and TRAIL are pro-apoptotic molecules that have been found to localize to the lytic granules of NK cells (Montel et al., 1995; Bossi and Griffiths, 1999; Monleon et al., 2001; Schmidt et al., 2009; Ghosh et al., 2010). FasL has been shown to be sorted to lysosomes through recognition of a proline-rich domain in its cytoplasmic tail (Blott et al., 2001). The lysine residues flanking this domain have been demonstrated to undergo mono-ubiquitination that is essential for targeting FasL to the lysosomes (Zuccato et al., 2007). It has been proposed that recruitment of kinases to the FasL proline-rich domain and phosphorylation of FasL is also critical for its targeting to lysosomes (Zuccato et al., 2007). One should keep in mind, however, that the phosphorylation of FasL might not be needed for its proper sorting, as the tyrosine residues identified in the aforementioned study lie in or near a motif (YxxI) that is known to target proteins to lysosomes, and mutations of such tyrosine residues often result in protein mis-sorting to the plasma membrane instead of lysosomes. The route by which TRAIL reaches the lytic granules has not been defined. Notably, TRAIL contains a motif that could be recognized by adaptor protein (AP) complexes, and thus mediate its transport from the TGN to the endo-lysosomal compartment. More experimentation is required to verify this hypothesis.

### Granulysin

Granulysin is expressed as two isoforms, 15 and 9 kDa, which differ in their function and cellular location. The 15 kDa isoform is present in vesicles negative for perforin or granzymes (Clayberger et al., 2012), although it remains to be elucidated whether it is present in the granules containing cytokines or is confined to another distinct pool of secretory vesicles. The larger isoform is not cytotoxic, but plays an important role in differentiation and activation of dendritic cells (Tewary et al., 2010; Clayberger et al., 2012). The 9 kDa isoform, generated by cleavage of the 15 kDa granulysin, localizes to lytic granules (Clayberger et al., 2012). It has been shown to be pro-inflammatory, and has a broad cytotoxic spectrum against gram-negative and gram-positive bacteria, fungi, parasites, and tumors (Stenger et al., 1998; Krensky and Clayberger, 2009; Clayberger et al., 2012).

Intriguingly, both granulysin isoforms have been demonstrated to act as chemoattractants for T cells, monocytes, and NK cells (Deng et al., 2005; Tewary et al., 2010). While the non-cytolytic form of granulysin could be easily imagined as a chemokine, the dual role of the cytotoxic isoform is puzzling. With its capacity to bind to membrane lipids and induce osmotic shock and apoptosis, it is not clear how the smaller form of granulysin could at the same time serve as a chemotactic molecule. One could speculate that the localized release of granulysin at the immunological synapse would achieve a high local concentration of the protein and ensure its cytolytic effect. On the other hand, the chemotaxis mediated by granulysin likely does not require high concentrations, especially if released together with other chemokines by activated NK cells, and its non-directional release would affect the entire area surrounding NK cells, thereby potentiating chemotactic activity. Additionally, granulysin induces the expression of chemokines (e.g., MIP-1α, RANTES, CCL2, and CCL8) and both pro- and anti-inflammatory cytokines (e.g., IL-1, IL-6, IL-10, and IFNα) (Deng et al., 2005). Therefore, granulysin appears to be a very important immunomodulatory molecule due to its ability to activate secretion of chemo- and cytokines, attract different immune cells, and induce apoptosis.

The lytic granules of NK cells also contain small peptides, namely **peptide LL-37 (cathelicidin), and defensins 1–3** (Agerberth et al., 2000; Obata-Onai et al., 2002; Chalifour et al., 2004).The release of these peptides results in direct anti-microbial effects, and contributes to activation of NK cells and surrounding cells (e.g., macrophages) through recognition of microbe-associated molecular patterns generated during the destruction of those pathogens. In rare cases of classical NK cell deficiency caused by GATA2 mutations, the patients are more susceptible not only for viral, but also for mycobacterial infections (Hsu et al., 2011), highlighting the importance of NK cells in providing anti-bacterial host defense. For an in-depth review of NK cell activities during microbial infections please refer to (Horowitz et al., 2011).

## Lytic granule membrane proteins

There is a variety of proteins that reside in lysosomal membranes and play different roles, including acidification of the lysosomal lumen, protein import, membrane fusion, and transport of degradation products to cytoplasm (Saftig and Klumperman, 2009). Recent proteomic studies provide an important insight into the understanding of the composition of proteins associated with the limiting membrane of lytic granules in NK or cytotoxic T cells (Casey et al., 2007; Schmidt et al., 2011). The function of the most abundant proteins in the lysosomal membrane, such as lysosome-associated membrane protein (LAMP)-1 and -2, or lysosomal integral membrane protein (LIMP)-2, is poorly described or simply unknown. The mutations in LAMP2 cause a hypertrophic cardiomyopathy and muscular dystrophy, known as Danon disease, that is believed to arise from aberration in autophagy, as muscle cells have abnormally large number of autophagic vacuoles; the patients, however, are not immuno-compromised (Nishino et al., 2000; Nishino, 2003). LAMP1 is widely used as a marker of NK cell degranulation (Alter et al., 2004; Aktas et al., 2009), and has been shown to accumulate in clusters at the immunological synapse that could be involved in membrane internalization (Liu et al., 2009), though the exact role the protein plays in NK cell biology has not been identified to date. Given that NK cells utilize their lysosomes to induce the death of target cells, one of the fundamental yet unresolved problems in NK cell biology is identifying and defining the importance of granule-specific membrane proteins that are required for exocytosis and/or biogenesis of the lytic granules.

## Lytic granule exocytosis

**Figure 2:**
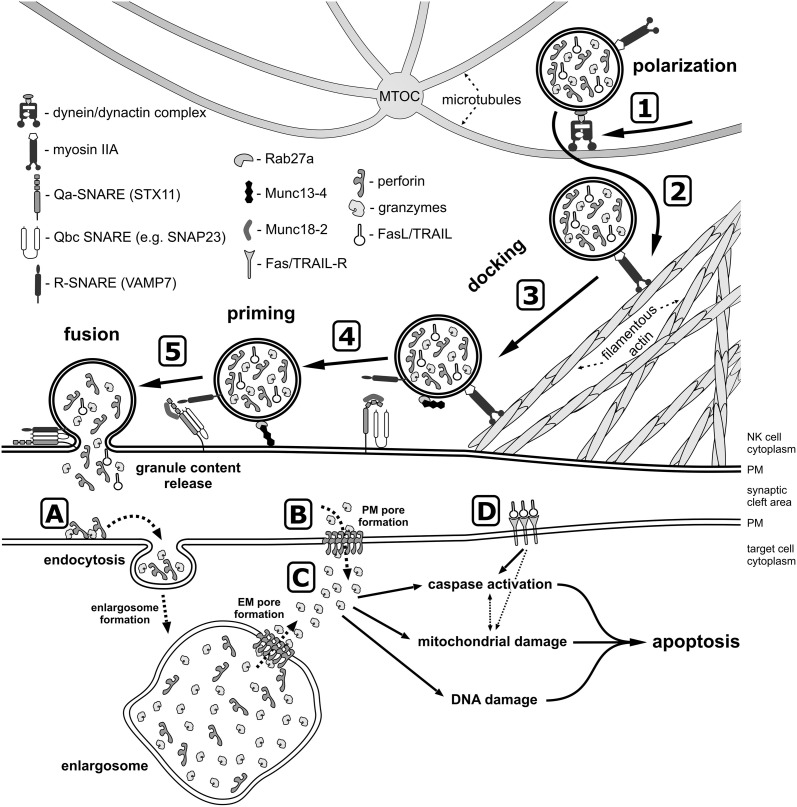
**A model of lytic granule exocytosis from human NK cell.** In response to the engagement of NK cell activating receptors and initiation of signaling cascades (not depicted), the lytic granules move along the microtubules toward the MTOC in the dynein-dynactin complex-dependent manner (1). The MTOC and the granules then polarize toward the NK-target cell contact area, where granules switch from microtubules to the filamentous actin network at the immunological synapse (2) and navigate through the cortical filamentous actin meshwork as a result of the actin motor protein myosin IIA activity (3). This allows the lytic granules to get into close proximity of the plasma membrane (PM), and dock at the membrane due to activity of Rab27a and Rab27a-mediated recruitment of Munc13-4, as well as through the recognition of syntaxin 11 (STX11) and Munc18-2, possibly by the R-SNARE protein(s) present at the lytic granule membrane. The docked granules are then primed (4) by Munc13-4 in response to calcium flux (not shown), likely by the Munc13-4-mediated switch of STX11 to an open conformation (by removal of Munc18-2), and/or by Munc13-4 forming an initial bridge between the granule membrane and the plasma membrane. Finally, the granule-associated R-SNARE protein(s) (e.g., VAMP7) form a complex with Q-SNARE proteins present on the plasma membrane (e.g., STX11 and SNAP23) (5), which allows for the fusion of vesicles with the plasma membrane and release of the granule content into the synaptic cleft at the immunological synapse. There are two paradigms describing the entry of perforin and granzymes into target cells. The internalization model (A) assumes that perforin and granzymes bind to the target cell plasma membrane and are endocytosed into the early endosome-like enlargosome. Following internalization, perforin would mediate formation of pores in the enlargosome membrane (EM), allowing granzymes to leak into the cytosol of the target cell. According to the plasma membrane (PM) pore formation model (B), perforin oligomerizes in the plasma membrane, disrupting its integrity thereby permitting granzymes to enter from the synaptic cleft into the target cell. After gaining access into the cell cytosol (C), granzymes start processing their targets, leading to apoptosis through activation of caspases, induction of mitochondrial damage, and DNA fragmentation. In addition, FasL and TRAIL from the lytic granules bind to their receptors on the target cell surface (D) and initiate apoptosis.

After receiving cues from the activating receptors and different signaling pathways, lytic granules in human NK cells first move along microtubules toward the MTOC, and then translocate with the MTOC toward the immunological synapse (Figure 2). This clustering process has been demonstrated to depend on the activity of minus-end-directed motor protein complex dynein-dynactin (Mentlik et al., 2010). Polarization of the lytic granules is not equivalent to a commitment to their secretion, and several more events are required before the granules fuse with the plasma membrane and their content is released into the synaptic cleft.

Although it has been shown that in human cytotoxic T cells and NK cells, the polarized MTOC docks at the plasma membrane and that phenomenon was postulated to be sufficient to facilitate a direct transfer of the lytic granules to the secretory area of the immunological synapse (Stinchcombe et al., 2006, 2011), lytic granules in NK cells have to navigate through the cortical actin network before they can fuse with the plasma membrane (Brown et al., 2011; Rak et al., 2011; Mace and Orange, 2012). High resolution imaging revealed a fine meshwork of filamentous actin covering the central area of the immunological synapse, with areas of hypodense actin regions spanning between 200 nm and 500 nm, which could allow for the lytic granules (average diameter 250–300 nm) to pass through (Brown et al., 2011; Rak et al., 2011). The navigation and transit of the granules through the clearances in the cortical filamentous actin is possible because of the actin motor protein, myosin IIA, and the disruption of myosin function renders human NK cells unable to release the lytic granules (Andzelm et al., 2007; Sanborn et al., 2009, 2011). An open question remains: how do the granules find the clearances in the actin meshwork? Do they dock directly at the openings in the actin cytoskeleton, or do they dock randomly at the cortical actin and then move laterally along the actin filaments until they find an opening? Is there a relationship between actin hypodense regions and position of the MTOC or microtubules at the immunological synapse?

An interesting issue pertains to how the areas of local hypodensity in the cortical actin meshwork are formed: through a local depolymerization of actin filaments, or perhaps through proteolytic degradation of the proteins involved in actin cytoskeleton rearrangements? The depolymerization and severing of actin filaments is mediated by cofilin (Pollard and Cooper, 2009), which has been demonstrated to be important in the secretion of glucose transporter-containing vesicles in muscle cells (Chiu et al., 2010). In the case of proteolytic remodeling of actin filaments, a possible candidate is calpain, a protease that has been shown to cleave ezrin, talin, and cortactin in several cell types (Shuster and Herman, 1995; Potter et al., 1998; Franco et al., 2004; Perrin et al., 2006). Calpain-mediated cleavage of ezrin or talin could release the actin cytoskeleton from LFA-1, as previously shown for T cells (Stewart et al., 1998), decreasing the rigidity of the actin meshwork in relation to the plasma membrane. The degradation of cortactin would prevent activation of the Arp2/3 complex and cause destabilization of the cortical actin network. In this regard, a cortactin homolog, HS1, has been shown to be critical for proper actin dynamics at the NK cell immunological synapse (Butler et al., 2008). Therefore, the areas of decreased filamentous actin density, required for the lytic granule release, could be formed in an active and regulated manner. Whether NK cell activating receptors could signal activation of cofilin or calpain remains to be elucidated. Another unresolved and challenging problem relates to whether the openings in the actin cytoskeleton at the immunological synapse are formed randomly or at a particular place. Are the hypodense regions juxtaposed to microclusters of NK cell surface receptors? Is there a correlation between receptor clustering, signaling, and formation of the actin cytoskeleton openings at the immunological synapse?

In addition, secretion of lytic granules requires the activity of docking and priming proteins, such as Rab27a, Munc13-4, and Munc 18-2 (Figure 2). Mutations in RAB27A, UNC13D and STXBP2 genes that encode these proteins result in severely impaired degranulation of NK cells without affecting the granule polarization to the immunological synapse, and lead to serious and often fatal immune disorders: Griscelli syndrome type 2, FHL type 3 and 5, respectively (Marcenaro et al., 2006; Cote et al., 2009; Wood et al., 2009; Zur Stadt et al., 2009; Meeths et al., 2010a,b; Rohr et al., 2010; Bryceson et al., 2012; Pagel et al., 2012) (Table 1). Interestingly, neither Rab27a nor Munc13-4 are present on the lytic granule surface of non-activated NK cells, but preferentially associate with lytic granules in response to engagement of different NK cell activating receptors (Wood et al., 2009). The small GTPase, Rab27a, is required for retention and cytoskeleton-dependent directional movement of lytic granules at the plasma membrane, as well as association of Munc13-4 with lytic granules in NK cells (Wood et al., 2009; Liu et al., 2010). The function of Munc13-4 is not fully elucidated. In cytotoxic T cells, Munc13-4 is involved in maturation of lytic granules and assembly of vesicles intended for exocytosis (Menager et al., 2007). Whether it plays the same role in human NK cells is not clear. The protein contains two C2 domains that bind Ca^2+^, and thus might function as a potential calcium sensor for NK cell exocytosis. Calcium flux is indispensable for lytic granule release, but not for granule polarization. Inositol-(1,4,5)-trisphosphate (IP3), a product of PLCγ activity, induces the release of Ca^2+^ from the intracellular store (i.e., endoplasmic reticulum) and aggregation of the ER calcium sensor STIM1, leading to activation of the calcium channel ORAI1 and calcium influx from an extracellular medium (Feske, 2010). Mutations in either ORAI1 or STIM1 result in impaired exocytosis, whereas lytic granule polarization to the immunological synapse remains unaffected in human NK cells (Maul-Pavicic et al., 2011). Similarly, disruption of function of the small GTPase Arf6, or silencing of its effector, phosphatidylinositol-4-phosphate-5-kinase (PI5KI type I), leads to deficient calcium flux and results in blockage of lytic granule exocytosis from NK cells (Galandrini et al., 2005; Micucci et al., 2008). In T cells, Munc13-4 has been also postulated to open conformation of a soluble N-ethylmaleimide-sensitive factor attachment protein receptor (SNARE) protein, syntaxin-11 (STX11), by removal of Munc18-2 (Elstak et al., 2011). Munc18-2 role in lytic granule release is likely related to its interaction with STX11. Munc18-2 is required for STX11 stabilization in NK cells: without functional Munc18-2, STX11 expression is severely decreased, and NK cell degranulation dramatically diminished (Cote et al., 2009).

**Table 1:** **Human diseases linked to lytic granules and defective NK cell function**.

**Disease**	**Gene mutated**	**Protein affected**	**Effect on the lytic granules**	**Lymphohistiocytosis**
Griscelli syndrome type 2	*RAB27A*	Rab27a	Impaired granule docking at the immunological synapse	Present
Chediak-Higashi syndrome	*CHS1/LYST*	LYST	Giant lysosomes, impaired granule exocytosis (unknown cause)	Present (the accelerated phase of the disease)
Hermansky-Pudlak syndrome type 2	*AP3B1*	β3A-subunit of the AP3 sorting complex (β3A-adaptin)	Enlarged lysosomes, impaired granule movement along the microtubules	Present
May-Hegglin anomaly	*MYH-9*	Myosin IIA	Impaired granule exocytosis due to inability to penetrate the cortical filamentous actin at the immunological synapse	Not present
Familial hemophagocytic lymphohistiocytosis type 2	*PRF1*	Perforin	Lytic granules are released, but their content is not delivered efficiently to target cells	Present
Familial hemophagocytic lymphohistiocytosis type 3	*UNC13D*	Munc13-4	Impaired granule docking and/or priming at the immunological synapse	Present
Familial hemophagocytic lymphohistiocytosis type 4	*STX11*	Syntaxin 11	Defective granule exocytosis due to impaired fusion of the lytic granules with the plasma membrane	Present
Familial hemophagocytic lymphohistiocytosis type 5	*STXBP2*	Munc18-2	Defective granule exocytosis due to impaired fusion of the lytic granules with the plasma membrane	Present

The final step of lytic granule release is the fusion of secretory lysosomes with the plasma membrane at the immunological synapse (Figure 2). Membrane fusion events are mediated by SNARE family of proteins, composed of Q- and R-SNARE subgroups, and often referred to as the membrane fusion machinery (Jahn and Scheller, 2006). Consequently, SNARE proteins are essential for degranulation of NK cells. For example, mutations of Qa-SNARE STX11 (causing FHL type 4; Table 1), or interfering with expression of R-SNARE VAMP4 or VAMP7, results in defective NK cell lytic granule exocytosis (Arneson et al., 2007; Bryceson et al., 2007; Marcet-Palacios et al., 2008; Krzewski et al., 2011). Since the functional SNARE complexes are comprised of one R-SNARE and either three (Qa, Qb, and Qc) or two (Qa, Qbc) Q-SNARE proteins (Jahn and Scheller, 2006), the complete membrane fusion complex (or complexes) is not fully defined in NK cells. Apart from STX11, possible candidate Qa-SNARE proteins include STX4 and STX6. STX4 participates in secretory granule exocytosis in mast cells, and likely interacts with VAMP7 during exocytosis of neutrophils (Mollinedo et al., 2006; Puri and Roche, 2006). STX 4, as well as STX6, known to interact with VAMP4 (Fujita-Yoshigaki et al., 2006), have been shown to be involved in TNFα secretion in macrophages (Pagan et al., 2003; Murray et al., 2005; Kay et al., 2006). Another SNARE candidate might be a Qbc-SNARE protein, SNAP23, that has been reported to be involved together with STX4 in degranulation of mast cells, macrophages, neutrophils and eosinophils (Pagan et al., 2003; Logan et al., 2006; Mollinedo et al., 2006; Puri and Roche, 2006). SNAP23 is also known to bind STX11 in B cells (Valdez et al., 1999).

Following the assembly of the SNARE complex, a fusion pore between the lytic granule and the plasma membrane is created. Interestingly, NK cell lytic granules have been recently shown to be capable of forming either fully-opened, or transient and incomplete pores. The full fusion of lytic granules results in rapid expulsion of granule content, while the partial fusion pore opening is associated with minimal content release and has been postulated to play a role in recycling of the lytic granule membrane (Liu et al., 2011). In addition, NK cells do not release all of the polarized lytic granules at once, only secreting a subset of the lytic granule pool following activation (Rak et al., 2011). This mode of lytic granule release could be related to the fact that cytotoxic lymphocytes are able to kill several target cells (Wiedemann et al., 2006; Bhat and Watzl, 2007). Alternatively, the partial release of the lytic granules could be caused by a mechanical barrier generated by the cortical actin underlying the immunological synapse. It has been estimated that less than 10% of the synapse area have openings in the actin meshwork sufficient to accommodate the granules (Brown et al., 2012). Therefore, the small area penetrable to the granules could be a limiting factor in their exocytosis.

## Death of the target cell

Following the release from the lytic granules, in the presence of physiological levels of Ca^2+^ (~1–1.3 mM) and neutral pH, perforin has the ability to insert itself into a membrane, oligomerize, and generate pores. The pores formed by human perforin appear to be heterogeneous, depending on local perforin concentration and composition of lipids in the membrane. Perforin can form either unstable, small arc-shaped pores, which most likely are incomplete channels assembled by oligomerization of perforin monomers, or generate large and stable ring-shaped pores (Praper et al., 2011a). Interestingly, the incomplete arc-shaped pores can induce a fusion between the inner and outer leaflet of the membrane and trigger externalization of phosphatidylserine (Metkar et al., 2011). The large, ring-shaped perforin channels in the membrane could be generated by maturation of the arc-shaped pores, or could form by an insertion of the fully assembled perforin complex into the membrane (Praper et al., 2011a). The size of pores generated by human perforin is estimated to be in 10–25 nm range, similar to 13–20 nm-diameter pore formed by 19–24 molecules of murine perforin (Law et al., 2010; Praper et al., 2011a).

Perforin is essential for the lytic activity of cytotoxic lymphocytes, as it is required for delivery of the apoptosis-inducing granzymes and their subsequent release into the cytoplasm of target cells (Keefe et al., 2005; Thiery et al., 2011). Even a small decrease in perforin expression correlates with marked reduction in NK cell cytotoxicity (Portales et al., 2003). The importance of perforin is further highlighted by the fact that perforin mutations lead to FHL type 2 and are linked to the autoimmune lymphoproliferative syndrome, and juvenile rheumatoid arthritis and macrophage activation syndrome (Grom et al., 2003; Molleran Lee et al., 2004; Voskoboinik et al., 2004, 2005b; Ishii et al., 2005; Villanueva et al., 2005; Clementi et al., 2006; Bryceson et al., 2007; Chia et al., 2012). These diseases are characterized by an increased number of overactive lymphocytes and/or histiocytes. They display similarities in terms of their clinical manifestations and immunologic mechanisms, one of them being the decreased or absent cytotoxic activity of NK cells due to lack of perforin and inability to deliver granzymes into the target cell cytosol. It has been proposed that the decreased cytolytic function of NK cells could be responsible for the lack of elimination of infected or activated cells, and thus contribute to the persistent inflammation and uncontrolled cell proliferation observed in lymphohistiocytic syndromes (Orange, 2008). Taking into consideration the function of NK cells during infections (Orange, 2002) and their ability to kill T cells, dendritic cells and, importantly, over-activated macrophages (Ferlazzo et al., 2002; Della Chiesa et al., 2003; Nedvetzki et al., 2007), it is reasonable to assume that the decreased ability to clear the infecting pathogen could lead to enhanced or persistent activation of T cells and macrophages. Consequently, the sustained activation of those cell types would result in increased production of pro-inflammatory cytokines and further activation of immune cells. A positive feedback loop of pro-inflammatory conditions would be generated, and the inflammatory response and cell proliferation of activated cells would continue because of the inability of NK cells to kill the activated cells and stop the amplification of the immune response in the aforementioned diseases. Likewise, other forms of the FHL or Griscelli syndrome 2 (see above and Table 1), characterized by defects in the lytic granule exocytosis, result in a similar disease pathogenesis as perforin deficiency; in all of these diseases, NK cells cannot efficiently deliver the apoptosis-inducing granzymes to target cells, and hence control the abnormal growth of lymphocytes and histiocytes.

How exactly granzymes are delivered into a target cell is an open debate, and data exist to support two paradigms. The classical model assumes that perforin, following its release from the lytic granules to the cleft at the immunological synapse, generates pores in the plasma membrane of the target cell. Granzymes are then able to diffuse through the perforin pore into the cytosol of the target cell, where they induce apoptosis through caspase-dependent and -independent mechanisms (Figure 2). In support of this model, the neutral pH and high calcium concentration at the immunological synapse would promote perforin oligomerization, and the ~20 nm size of the perforin pores would allow granzymes to easily pass through (Dourmashkin et al., 1980; Voskoboinik et al., 2010; Praper et al., 2011a). The second model assumes that perforin and granzymes are internalized by endocytosis (as a result of the membrane repair response or binding of the perforin-serglycin-granzyme macromolecular complex to MPR receptors on the cell surface). Following the internalization, perforin would mediate disruption of endosomal membranes, allowing granzymes to leak into the cytosol of the target cell (Figure 2). Supporting this paradigm, perforin has been shown to induce membrane invaginations, formation of vesicles, and trigger endocytosis-like events (Keefe et al., 2005; Thiery et al., 2010; Praper et al., 2011b). Moreover, granzymes have been found to be endocytosed in a clathrin-dependent manner by target cells, and their subsequent cytosolic delivery required perforin (Froelich et al., 1996b; Edwards et al., 1999; Keefe et al., 2005; Thiery et al., 2010). A recent report also demonstrated that following endocytosis of perforin and granzyme B to early endosome-like compartment (positive for EEA-1, but significantly enlarged, and hence termed enlargosome), perforin was able to oligomerize in the membranes of the enlarged endosomes, due to lack of acidification of those structures, and thus mediate release of granzyme B into the cytoplasm (Thiery et al., 2011).

In general, the activity of granzymes results in enforcing apoptosis through generation of reactive oxygen species, mitochondrial damage, and DNA fragmentation. These effects can be induced in caspase-dependent and/or -independent manner. The caspase-dependent activities involve processing of different caspases. Granzyme B, for instance, has been shown to cleave and activate a range of caspases, both *in vitro* and *in vivo* (Table 2), thus inducing apoptosis through direct activation of caspases (Medema et al., 1997; Barry et al., 2000; Adrain et al., 2005). The caspase-independent initiation of apoptosis by granzyme B and K includes cleavage of a pro-apoptotic protein, Bid, which leads to mitochondrial outer membrane permeabilization, release of cytochrome C, and activation of the mitochondrial pathway (Heibein et al., 1999; Barry et al., 2000; Sutton et al., 2000; Pinkoski et al., 2001; Adrain et al., 2005; Zhao et al., 2007a). Other caspase-independent functions of granzymes include processing of proteins that are involved in activation or sensing DNA damage (Table 2). For example, granzyme A and K cleave components of the SET complex, which keeps NM23-H1 and TREX1 nucleases inactive. Following the release from the SET complex, these nucleases induce single-stranded DNA damage (Beresford et al., 2001; Fan et al., 2002, 2003a,b; Chowdhury et al., 2006; Zhao et al., 2007b). Additionally, granzyme A, B, and M cleave DNA repairing enzymes (e.g., PARP, DNA-PKcs, Ku70) (Froelich et al., 1996a; Casciola-Rosen et al., 1999; Lu et al., 2006; Zhu et al., 2006, 2009), which interferes with the ability of the cell to remedy the DNA damage, and consequently increases the amplitude of that pro-apoptotic effect.

**Table 2:** **Granzymes and their substrates**.

**Name**	**Induction of apoptosis**	**Substrates**	**References**
Granzyme A	Caspase-independent	Mitochondrial respiratory complex I protein (NDUFS3); SET complex (SET, Ape1, HMG2); poly-ADP-ribose polymerase (PARP); Ku70; lamins; histones	Beresford et al., 2001; Zhang et al., 2001a,b; Fan et al., 2002, 2003a,b; Martinvalet et al., 2005, 2008; Chowdhury et al., 2006; Zhu et al., 2006, 2009
Granzyme B	Caspase-dependent and -independent	Caspase-3, -7, -8, and -10; Bid; tubulin α; Rho-associated coiled coil-containing protein kinase II (ROCK II); lamin B; inhibitor of caspase-activated DNase (ICAD); DNA-dependent protein kinase catalytic subunit (DNA-PKcs); PARP	Froelich et al., 1996a; Medema et al., 1997; Heibein et al., 1999; Barry et al., 2000; Sutton et al., 2000; Pinkoski et al., 2001; Sharif-Askari et al., 2001; Zhang et al., 2001a; Adrain et al., 2005; Sebbagh et al., 2005; Goping et al., 2006; Andrade et al., 2007
Granzyme H	Caspase-dependent and -independent	Caspase-3 (indirect); adenoviral 100K assembly protein; does not cleave ICAD or Bid	Andrade et al., 2007; Fellows et al., 2007; Hou et al., 2008
Granzyme K	Caspase-independent	Bid; SET complex (HMG2, Ape1, SET); p53	Zhao et al., 2007a,b; Hua et al., 2009
Granzyme M	Caspase-dependent and -independent	Nucleolar protein nucleophosmin; Fas-associated protein with death domain (FADD); survivin/BIRC5; heat shock protein TRAP1; ICAD; PARP; ezrin; tubulin α; serpin PI-9/B6	Mahrus et al., 2004; Lu et al., 2006; Hua et al., 2007; Bovenschen et al., 2008; Hu et al., 2010; Wang et al., 2012

While the activities of granzymes A and B are well described, there is less information about the function of granzymes H and M. Granzyme H has been shown to induce mitochondrial damage and DNA fragmentation. Those processes were reported to be both caspase-dependent, involving phosphatidylserine externalization, the release of cytochrome C and caspase-3 activation, and caspase-independent, not involving the cleavage of Bid protein, or release of cytochrome C (Fellows et al., 2007; Hou et al., 2008). The exact mechanism of granzyme M action is not fully elucidated, and there is controversy regarding the pathway used by granzyme M to cause apoptosis of target cells. Granzyme M has been reported to initiate caspase-independent death of target cells, similarly to granzymes A and K. In contrast to these two granzymes, however, granzyme M does not induce generation of reactive oxygen species or cytochrome release, indicating that the mitochondrial damage pathway is not involved in granzyme M-mediated target cell apoptosis (Kelly et al., 2004; Cullen et al., 2009). Other reports have shown that granzyme M has the ability to activate caspase-dependent cell death by activation of caspase 8 and 9, and induction of DNA fragmentation, as well as generation of reactive oxygen species and release of cytochrome C (Lu et al., 2006; Hua et al., 2007; Hu et al., 2010; Wang et al., 2012).

Regardless of the nuances in defining the precise roles of individual granzymes, one can conclude that granzymes display a broad spectrum of activities and trigger many apoptotic pathways to ensure that the target cells die following the lytic granule exocytosis by NK cells. Interestingly, no disorder arising from a single granzyme deficiency has been reported in humans to date. A possible explanation could be that granzymes share multiple substrates (Table 2) and utilize several pathways to induce the cell death, and hence the disruption of an individual granzyme function likely would not block the capacity of NK or T cells to induce the apoptosis, as long as the granzymes are delivered to the target cell cytosol.

How are the other lytic granule molecules involved in NK cytotoxic function? In the case of granulysin, the literature points to an intricate mode of action. The crystal structure of granulysin reveals that the surface of the molecule has a positive charge (Anderson et al., 2003). Therefore, the binding of granulysin to target membranes is most likely mediated by an electrostatic interaction. In support of this concept, no specific receptor for granulysin has been identified to date. Of note, however, is the fact that granulysin has been postulated to activate a G-coupled protein receptor (GPCR) and TLR4, at least in a mouse model (Tewary et al., 2010), but the identity of GPCR has not been determined, and it is not known whether granulysin could directly bind the GPCR or form complexes with negatively-charged molecules and thus activate pattern recognition receptors, such as TLR4.

Granulysin binding to and the fracturing of a target cell plasma membrane induces a flux of calcium and potassium. Blockage of the ion flux protects from cell lysis (Okada et al., 2003), underlining the importance of this step in granulysin function. The granulysin-mediated increase of intracellular calcium could contribute to the mitochondrial damage and induction of apoptosis. Indeed, granulysin has been shown to damage the mitochondrial membrane in the presence of calcium, and cause the release of cytochrome C and production of reactive oxygen species (Kaspar et al., 2001; Okada et al., 2003). Furthermore, granulysin contributes to activation of caspase 3 (Kaspar et al., 2001), which further potentiates mitochondrial damage and provides a positive feedback loop for activation of apoptosis. A recent report demonstrated that granulysin also induces damage of the endoplasmic reticulum in a caspase 7-dependent manner (Saini et al., 2011). Since the ER serves as a calcium store, the disruption of ER integrity by granulysin could lead to an increase of intracellular calcium and trigger apoptosis. In addition, granulysin has been shown to induce permeabilization of lysosomal membranes, resulting in the release of lysosomal content to the target cell cytoplasm and induction of apoptosis (Zhang et al., 2009). All these data emphasize a broad spectrum of cytotoxic effects mediated by granulysin.

Following the fusion of the lytic granules with the plasma membrane, FasL and TRAIL are exposed on the NK cell surface (Bossi and Griffiths, 1999; Johnsen et al., 1999; Bryceson et al., 2005). Ligation of TRAIL receptors or Fas on the cell surface by TRAIL or FasL, respectively, leads to trimerization of the receptors and subsequent formation of the death-inducing signaling complex, activation of the caspase cascade, and induction of apoptosis [reviewed in (Falschlehner et al., 2007; Lavrik and Krammer, 2012)]. Additionally, TRAIL has been demonstrated to induce permeabilization of lysosomal membranes, which results in the release of cathepsin B into the cytoplasm of target cells, and activation of apoptosis through the mitochondrial pathway (Werneburg et al., 2012).

Despite their important role in induction of apoptosis, the biology of FasL or TRAIL secretion in NK cell cytotoxicity has not been extensively studied. Interestingly, the involvement of these two proteins in the killing of target cells may depend on the maturation stage of NK cells. Immature (defined as CD161^+^ CD56^−^) NK cells appear to kill the target cells through a TRAIL-, but not FasL-dependent pathway, while the mature (i.e., CD161^+^ CD56^+^) NK cells utilize both TRAIL and FasL to induce apoptosis of target cells (Zamai et al., 1998). How exactly this phenomenon is regulated is not clear, as the mRNA for FasL and TRAIL is detectable in all NK cell populations. It is possible that FasL could be expressed at the protein level later than TRAIL, or TRAIL expression level could be much higher than FasL. More detailed analysis of FasL and TRAIL expression regulation in NK cells is needed to resolve that issue.

## Concluding remarks

With the current progress in resolution of imaging techniques and better understanding of the events leading to and/or required for the exocytosis of lytic granules from human NK cells, we have gained significant insight into the complex processes governing NK cell cytotoxicity. Still, there are many questions that remain unanswered. For example, what other motor proteins, especially microtubule-associated, are involved in lytic granule exocytosis? A microtubule motor protein, kinesin-1, has been recently demonstrated to be important in lytic granule release from T cells (Kurowska et al., 2012), and the bi-directional movement of granules along the microtubules has to involve some of the plus-end microtubule motor proteins. Is the biogenesis and maturation of lytic granules the same in NK and T cells, given that NK cells express many of the granule components constitutively and the granules are pre-formed in the cells without a need for activating stimuli? Are all proteins essential in lysosomal biogenesis critical for NK cell lytic granule biogenesis and/or exocytosis? Some of the lysosomal storage disorders, e.g., Chediak-Higashi syndrome or Hermasky-Pudlak syndrome type 2, are characterized by defective NK cell function and immunodeficiency (Table 1), but the function of the genes mutated in these diseases (LYST and AP3BP1) has not been fully elucidated in NK cell biology. For thorough reviews on how different disorders affect NK cell function, please refer to (Orange, 2006) and (Wood et al., 2011). Comprehensive understanding of the mechanisms regulating release of lytic granules from NK cells and identification of the role of components critical for NK cell degranulation will aid in the development of approaches that enhance the immunotherapeutic value of NK cells in treating cancer, pathogen-induced diseases and disorders caused by the impairment of cytotoxic lymphocyte function.

### Conflict of interest statement

The authors declare that the research was conducted in the absence of any commercial or financial relationships that could be construed as a potential conflict of interest.
